# Suppressed endothelin-1 by anti-VEGF therapy is important for patients with BRVO-related macular edema to improve their vision

**DOI:** 10.1186/s13167-016-0066-2

**Published:** 2016-08-24

**Authors:** Teruyo Kida, Josef Flammer, Hidehiro Oku, Seita Morishita, Masanori Fukumoto, Hiroyuki Suzuki, Katarzyna Konieczka, Tsunehiko Ikeda

**Affiliations:** 1 Department of Ophthalmology, Osaka Medical College, 2-7 Daigaku-machi, Takatsuki, Osaka 569-8686 Japan; 2 Department of Ophthalmology, University of Basel, Basel, Switzerland

**Keywords:** Endothelin-1, Anti-VEGF, Branch retinal vein occlusion (BRVO), Macular edema

## Abstract

**Background:**

Branch retinal vein occlusion (BRVO) commonly occurs at the arteriovenous crossing in the unilateral eye, and cardiovascular diseases can be risk factors of BRVO. However, the pathomechanism leading to BRVO is not yet clear. In addition to mechanical compression, the vein might locally constrict due to an altered biochemical environment, such as an increase in the concentration of endothelin-1 (ET-1). We evaluated changes in ET-1 following injection of intravitreal bevacizumab (IVB), which is the anti-vascular endothelial growth factor (VEGF) agent with the longest serum half-life, to determine the effect on BRVO-related macular edema.

**Methods:**

Twenty consecutive patients with BRVO-related macular edema (10 males, 10 females; age range 56–83 years) who visited our hospital were included in this prospective study. Visual acuity (VA); central retinal thickness (CRT), determined by macular optical coherence tomography (OCT); and plasma ET-1 levels were obtained before IVB treatment and 1 month later.

**Results:**

Patients had hypertension (80 %), dyslipidemia (50 %), diabetes mellitus (35 %), or collagen disease (5 %). Mean CRT was significantly decreased from 673.0 ± 327.8 to 388.2 ± 155.0 μm (*P* = 0.0007), and mean VA was significantly improved after IVB (*P* = 0.0239). Mean plasma ET-1 was significantly decreased from 1.272 ± 0.451 to 1.095 ± 0.316 pg/mL (*P* = 0.0238); however, the plasma ET-1 level was increased in all five patients who did not show improved VA after IVB.

**Conclusions:**

In patients with BRVO-related macular edema, anti-VEGF therapy leads to an expected reduction in ET-1 levels; however, the ET-1 level was found to increase in some patients; this is clearly related to less improvement of VA after anti-VEGF therapy.

**Trial registration:**

University hospital Medical Information Network (UMIN) Center UMIN000013236. Registered 10 October, 2012.

## Background

Branch retinal vein occlusion (BRVO) was first reported in 1896 [1]. It is known that the major risk factors for BRVO include increasing age, systemic hypertension, dyslipidemia, and coexisting cardiovascular diseases [2, 3]. Therefore, affected patients are also at higher risk for future cardiovascular events. The pathomechanism leading to BRVO is not yet clear. BRVO commonly occurs at the arteriovenous crossing. It is assumed that the diseased artery mechanically compresses the vein. However, it has been reported that the retinal vein seems to run deep under the artery at the crossing in eyes with arterial overcrossing, and the venous lumen often appears to be preserved, even at the arteriovenous crossing, as observed by thin sectioning with optical coherence tomography (OCT) in patients with acute BRVO [4]. In addition, there is another possible involvement that the vein locally constricts actively due to an altered biochemical environment, such as locally increased endothelin-1 (ET-1) concentration. The occlusions seem to be induced by an interaction between local and systemic factors; on the one hand, BRVOs generally occur unilaterally, while on the other hand, retinal venous pressure is also simultaneously increased in the clinically unaffected contralateral eye [5, 6].

Macular edema is the major vision-threatening complication associated with BRVO, and it often causes vision decline in patients with this disease. The congestion of the vein can lead to local hypoxia, thereby increasing vascular endothelial growth factor (VEGF), which in turn contributes to macular edema [7, 8]. Anti-VEGF therapy leads to the rapid reduction of macular edema and an improvement in visual function [9]. However, anti-VEGF therapy does not improve visual acuity (VA) in every patient with BRVO, although a regression in macular edema is present after performing the therapy. It indicates that factors other than VEGF are potentially involved as well.

One important candidate is endothelin [10]. ET-1 is a potent vasoconstrictor, and it regulates the blood-retinal barrier, stimulates the growth and migration of cells, and regulates axoplasmic transport. It is essential for the maintenance of cardiovascular homeostasis [11]. While ET-1 is mainly produced by vascular endothelial cells under physiological conditions, it can be produced by any other cell under pathological conditions, such as hypoxia or inflammation. The increase of VEGF points to a local hypoxia [12]. As a result, VEGF, ET-1, or erythropoietin and other molecules are overexpressed. The overexpression of VEGF hints at a simultaneous overexpression of ET-1, and most probably, both VEGF and ET-1 are involved in the pathogenesis of macular edema. There is a multifaceted relationship between VEGF and ET-1. They are opponents in terms of regulation of the size of vessels; while VEGF has vasorelaxing activity, ET-1 is a potent vasoconstrictor. However, they act synergistically on the blood-retinal barrier, and they both weaken the barrier. In addition, a stimulatory interaction exists between VEGF and ET-1, affecting gene expression and secretion in vascular cells. Therefore, reducing VEGF normally also reduces the concentration of ET-1 and vice versa.

In this prospective study, we investigated whether an anti-VEGF therapy could influence the concentration of ET-1 which had never been measured before and after anti-VEGF therapy and, if so, whether changes in the ET-1 levels are somehow related to the recovery of VA in patients with BRVO-related macular edema. With this hypothesis, we collected consecutive patients with acute BRVO-related macular edema prospectively and measured their plasma ET-1 levels before and after injecting intravitreal bevacizumab (IVB), which has the longest serum half-life among the current available anti-VEGF drugs [13].

## Methods

This prospective study was approved by the Institutional Review Board (IRB) at Osaka Medical College, and it adhered to the tenets of the Declaration of Helsinki. For this prospective study, consecutive patients with untreated macular edema secondary to unilateral BRVO who visited the outpatient clinic of Ophthalmology at Osaka Medical College Hospital between November 2012 and April 2013 were enrolled.

The inclusion criteria of this study were as follows: (1) symptomatic BRVO with macular edema; (2) a foveal thickness greater than 250 μm, as measured by OCT at the initial visit; and (3) macular edema secondary to BRVO, which had never been previously treated. Diagnosis of BRVO was based on the findings from fundus examinations and fluorescein angiographies. Eyes with any coexisting ocular diseases, such as age-related macular degeneration, diabetic retinopathy, hypertension retinopathy, or uveitis, were excluded.

At the initial visit, medical history, including disease durations, was obtained from each patient through a medical interview. At the initial examination, each patient underwent comprehensive ophthalmic examinations, which included the measurement of best-corrected VA using a Landolt chart and fundus biomicroscopy with a non-contact lens. Digital fundus photography, fluorescein angiography, and OCT examination were also performed after pupil dilation. The BRVO present in each case was classified as ischemic if the fluorescein angiography revealed more than 10 disc areas of retinal non-perfusion.

All patients were treated by injection of IVB after obtaining their informed consent. At each visit before IVB and 1 month after IVB, each patient underwent a comprehensive ophthalmologic examination, including measurement of best-corrected VA, color fundus photography, and OCT examination; moreover, blood was collected to measure the patient’s plasma ET-1 level. Plasma ET-1 levels were measured using an enzyme-linked immunosorbent assay (ELISA) kit (Takara Bio, Shiga, Japan).

All values are presented as the mean ± standard deviation. For statistical analysis, VA measured with a Landolt chart was converted to the logarithm of the minimum angle of resolution (logMAR). Central retinal thickness (CRT) measured with OCT was determined as an average retinal thickness in a 1-mm-diameter circular region at the fovea. Paired *t* tests were used to evaluate changes in the best-corrected VA (logMAR), CRT, and plasma ET-1 levels.

## Results

In this study, we report on 20 eyes from 20 different patients with BRVO-related macular edema that received treatment with IVB. No ischemic BRVO was observed in any of the patients at the first visit, and none of the patients converted to ischemic after receiving the IVB treatment. Table 1 shows the demographics and characteristics of changes in VA, CRT, and plasma ET-1 levels before IVB and 1 month after IVB for each patient. Mean age was 66.1 ± 7.6, ranging from 56 to 83 years. The patients had additional systemic diseases as follows: 16 (80 %) had hypertension, 10 (50 %) had dyslipidemia, 7 (35 %) had diabetes mellitus without retinopathy, and 1 (5 %) had collagen disease.

**Table 1:** Characteristics of patients with macular edema associated with branch retinal vein occlusion treated by intravitreal injection of bevacizumab

Patients	Age (years)	Gender	General condition	Before initial injection		1 month after initial injection		Plasma ET-1 level (pg/mL) before and after the treatment	
				VA	CRT (μm)	VA	CRT (μm)	Pre	Post
1	67	M	HT	0.222	577	0.046	212	1.763	0.982
2	68	F	DM, HT	1	953	0.398	236	1.057	0.568
3	57	M	HT, HL	0.398	692	0.155	364	1.057	0.982
4	71	M	HT	0.699	577	0.824	503	1.098	1.408
5	83	F	HT	1	489	0.523	391	2.121	1.388
6	61	F	HT, HL	1.155	1085	0.398	523	0.965	0.848
7	70	M	HT	0.398	466	0	198	1.662	1.005
8	61	F	HT, HL	0.523	340	0.699	351	0.848	1.204
9	62	M	DM	0.398	720	0.398	662	1.686	1.467
10	51	F	HT, HL	0.523	404	0	213	1.105	1.090
11	67	M	HT, HL	1.097	1093	1.097	631	1.617	1.602
12	78	M	DM, HT, HL	0.523	529	0.699	488	0.568	0.613
13	69	F	CD	1.523	1731	1.523	412	1.602	1.475
14	62	F	DM, HL	0.523	670	0.523	567	0.940	0.832
15	56	M	HL	0.398	562	0.398	540	1.456	1.041
16	68	F	DM, HT	0.699	651	0.824	433	0.613	0.737
17	67	F	HT, HL	0.398	612	0.398	160	1.268	1.244
18	75	M	DM, HT, HL	1	442	0.699	408	2.038	1.602
19	67	M	HT	0	507	0.097	210	0.762	0.864
20	61	F	DM, HT	0.699	359	0.398	261	1.204	0.940
Mean ± SD	66.1 ± 7.6			0.66 ± 0.37	673.0 ± 327.8	0.51 ± 0.38	388.2 ± 155.0	1.272 ± 0.451	1.095 ± 0.316

*M* male, *F* female, *DM* diabetes mellitus, *HT* systemic hypertension, *HL* hyperlipidemia (dyslipidemia), *CD* collagen disease, *VA* visual acuity in logarithm of the minimum angle of resolution (logMAR) fashion, *CRT* central retinal thickness

**Fig. 1:**
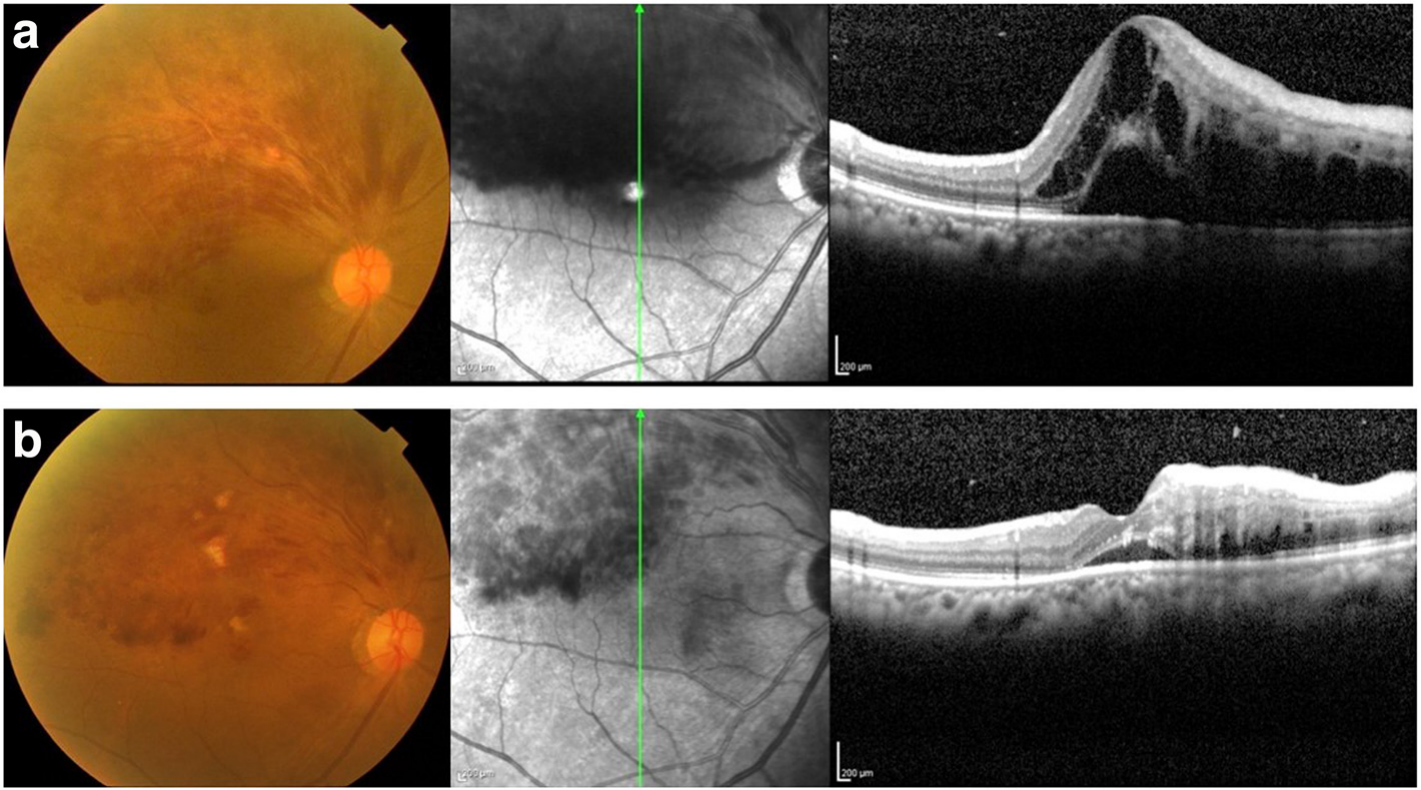
A case of 68-year-old female, BRVO with macular edema (patient 2 in Table 1). **a** *Upper*: pre-treatment; **b** *lower*: 1 month after intravitreal injection of bevacizumab (IVB). Macular edema was dramatically decreased, and the visual acuity was improved from 2/20 to 8/20 1 month after IVB. Plasma ET-1 level was decreased from 1.0567 to 0.568 pg/mL

**Fig. 2:**
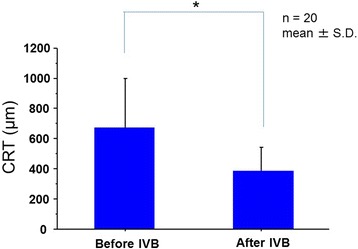
Changes in central retinal thickness (CRT) before and after the treatment. Mean CRT was significantly decreased after the treatment (*P* = 0.0007, paired *t* test)

**Fig. 3:**
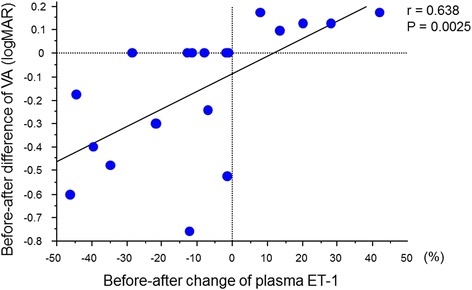
A regression line of changes in ET-1 and VA. Before-after change of plasma ET-1 was calculated as follows: (ET-1 level after IVB − ET-1 level before IVB)/ET-1 level before IVB × 100 (%). There was a correlation between changes in ET-1 level and VA before and after IVA (*r* = 0.638, *P* = 0.0025)

Before treatment, all eyes showed retinal hemorrhage and macular edema associated with BRVO. Figure 1 shows the fundus photos and the OCT findings for a case of macular edema secondary to BRVO before IVB treatment and 1 month after treatment. One month after an initial IVB injection, reduction of the macular edema was observed in 19 (95 %) eyes. Mean CRT was significantly decreased from 673.0 ± 327.8 μm before treatment to 388.2 ± 155.0 μm 1 month after the initial injection (*P* = 0.0007, Fig. 2). Mean VA was significantly improved from 0.66 ± 0.37 before the treatment to 0.51 ± 0.38 1 month after treatment (*P* = 0.0239). The mean plasma ET-1 level was significantly decreased after IVB from 1.272 ± 0.451 to 1.095 ± 0.316 pg/mL (*P* = 0.0238). Changes in the plasma ET-1 level in the 20 patients were correlated with changes in VA (*P* = 0.0025, Fig. 3).

**Fig. 4:**
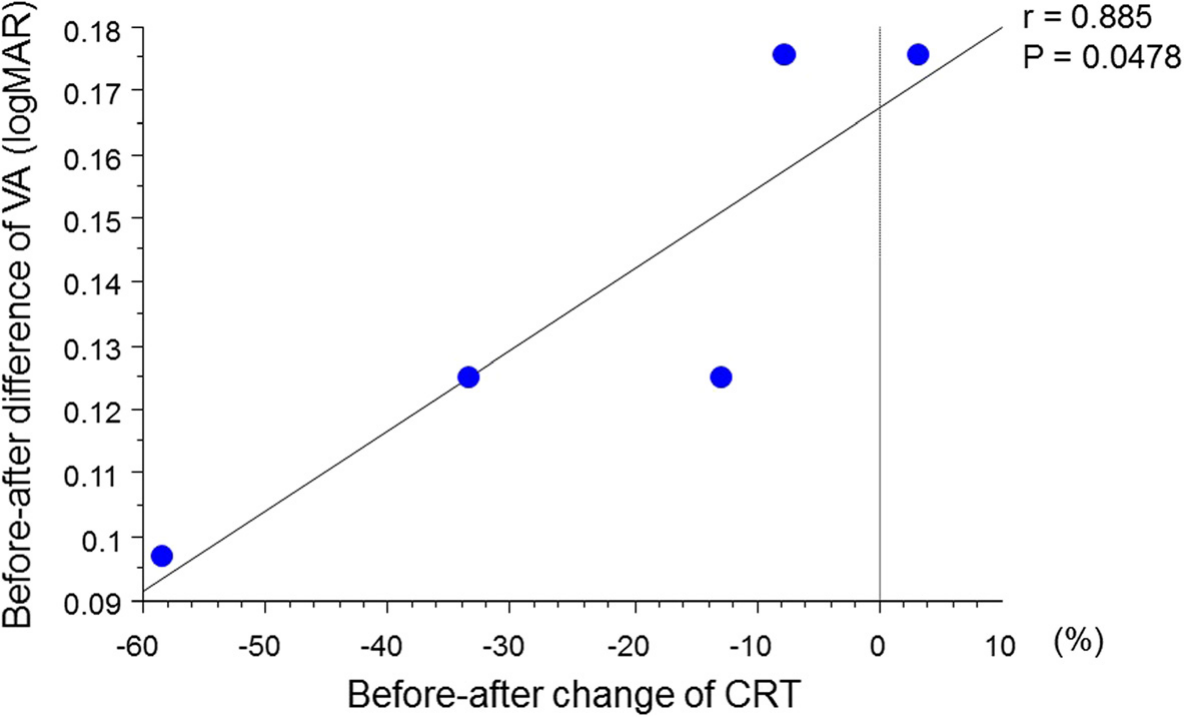
A regression line of changes in CRT and VA in five patients with increased ET-1 after IVB. Before-after change of CRT was calculated as (CRT after IVB − CRT before IVB)/ET-1 level before IVB × 100 (%). There was a correlation between changes in CRT and VA (*r* = 0.885, *P* = 0.0478)

**Fig. 5:**
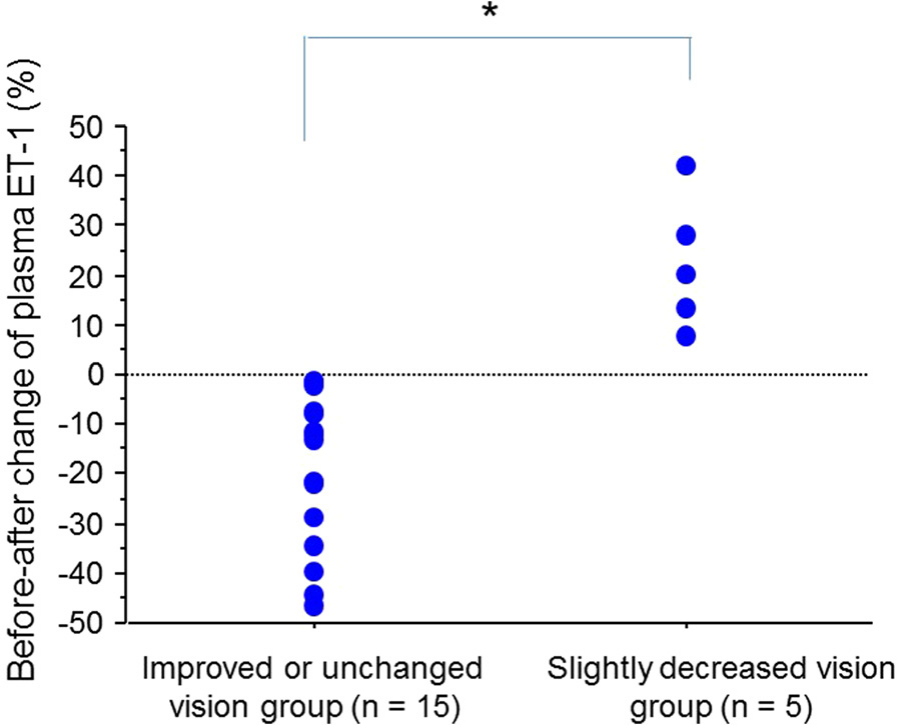
Before-after changes of plasma endothelin-1 (ET-1) level. Before-after change of plasma ET-1 (%) was calculated as follows: (ET-1 level after IVB − ET-1 level before IVB)/ET-1 level before IVB. Every patient who had visual improvement or maintained VA after anti-VEGF therapy showed significantly decreased plasma ET-1 (*P < 0.0001, unpaired *t* test)

Of the 20 eyes, VA was improved or maintained in 15 (75 %) eyes 1 month after the IVB treatment. In these 15 patients, the plasma ET-1 level was significantly decreased after IVB (*P* = 0.0007). On the other hand, the plasma ET-1 level was increased in all five patients who showed slightly decreased VA after IVB. In these patients with increased ET-1 level after IVB, changes in CRT were correlated with changes in VA (*P* = 0.0478, Fig. 4). To compare changes in plasma ET-1 levels before and after the IVB treatment in improved or maintained vision patients (*n* = 15) and the other patients (*n* = 5), we calculated before-after changes of plasma ET-1 levels (%). A significant difference in changes of plasma ET-1 level before and after IVB was observed between these two subgroups (*P* < 0.0001, unpaired *t* test, Fig. 5).

## Discussion

In this prospective study, a local anti-VEGF treatment for BRVO-related macular edema was applied to the eyes of 20 patients. One month later, CRT was significantly reduced in 19 patients (95 %), VA was improved or remained stable in 15 patients (75 %), and VA slightly decreased in 5 patients (20 %). On average, the plasma ET-1 level was significantly decreased after IVB. In addition, the ET-1 response showed a remarkably and statistically correlated change of VA. In patients with increased ET-1 level after IVB, there was a correlation between changes in CRT and VA.

It is known that a reduction of VEGF normally also leads to a reduction in ET-1 [14], and this reduction can be explained by the stimulatory interaction between VEGF and ET-1; however, the increase in the plasma ET-1 level in patients with slightly decreased VA was a surprising finding. The mechanism of this is unclear from our study, and we only have a hypothetical explanation for this result. Under hypoxia, nature produces VEGF in an attempt to improve the oxygen supply; thus, it is feasible that, in some cases, the reduction of the VEGF level, while also reducing macular edema by improving the blood-retinal barrier, can further increase local hypoxia. This might also impede improved VA or stimulate the production of ET-1. This stimulation may overcome the inhibitory effect of the VEGF drop. Here, we would like to emphasize, again, that what is going on locally is obviously important. Nevertheless, the systemic level of ET-1 may provide some indirect insight. The reactive increase in the ET-1 level after treatment with the anti-VEGF agent raises the question as to whether the combination of an anti-VEGF therapy with an anti-ET-1 treatment could improve visual prognosis in these patients. Primarily, the goal of the anti-VEGF therapy results should be on the macular edema, not on the VA, because the VA results are dependent on many other factors as well. In experimental retinal vein occlusion, ET-1 blockers were found to improve retinal circulation [15, 16]. In patients with retinal vein occlusion, calcium channel blockers for systemic hypertension were found to reduce retinal venous pressure [17, 18]. This partly reduces the effect of ET-1.

The current prospective study has some limitations: its sample size was small and its follow-up duration was short. Moreover, we measured the ET-1 level in plasma, not in intraocular fluid. As under clinical conditions, a quantification of ET-1 in the retina is not possible; therefore, we measured the plasma ET-1 level using an indirect approach. The plasma ET-1 level only provides indirect and approximate information about the local ET-1 level in the retina. Actually, Iannaccone et al. [19] reported that plasma ET-1 levels in patients with central retinal vein occlusion were significantly higher than those in the normal subjects. Sin et al. [20] measured aqueous ET-1 levels in patients with BRVO, and their ET-1 levels in aqueous humor were more than 10 times higher than the plasma ET-1 levels in our data. These results indicate the possibility that the vascular endothelial cells—and potentially any other cells in the eye—could produce ET-1 under pathological conditions. In addition, in their paper about plasma ET-1 levels before and after systemic treatment of bevacizumab for patients with metastatic colorectal cancer, Dirican et al. [21] found a significant decrease in serum ET-1 levels after treatment with bevacizumab. The effects on the retinal vasculature might be somewhat varied; however, anti-VEGF therapy influences ET-1. In this prospective study, we indicated that ET-1 is possibly involved in the pathogenesis of BRVO, and some BRVO patients whose VA was not improved after IVB showed increased plasma ET-1 levels even though intraocular VEGF was suppressed by anti-VEGF therapy. If we saw a patient with BRVO-related macular edema whose ET-1 level was not decreased after an initial injection of anti-VEGF agent, we could consider the additional injection.

## Conclusions

In conclusion, a local anti-VEGF therapy for macular edema in patients with BRVO led to an increase in the plasma ET-1 level in some patients without visual improvement. The causal relationship and the therapeutic consequences are not yet clear. However, ET-1 suppressed by anti-VEGF therapy is thought to be important for patients with BRVO-related macular edema, and this treatment approach could be used to improve their vision. The question remains open in how far is the connection between the ocular VEGF production, the influence of anti-VEGF therapy, and the plasma ET-1 concentration in the decrease of the CRT and VA improvement in patients with BRVO.
